# The Interleukin-33/ST2 axis promotes glioma mesenchymal transition, stemness and TMZ resistance via JNK activation

**DOI:** 10.18632/aging.102707

**Published:** 2020-01-29

**Authors:** Lin Lin, Yang Li, Mingli Liu, Qingbin Li, Quan Liu, Ruiyan Li

**Affiliations:** 1 Department of Neurosurgery, Second Affiliated Hospital of Harbin Medical University, Harbin 150086, China; 2 Chinese Glioma Cooperative Group (CGCG), Beijing 100050, China; 3 Neuroscience Institute, Heilongjiang Academy of Medical Sciences, Harbin 150086, China; 4 Southern University of Science and Technology, Shenzhen 518055, China

**Keywords:** IL-33, EMT, stemness, glioma, temozolomide

## Abstract

IL-33 is an important member of the IL-1 family which has pleiotropic activities in innate and adaptive immune responses. Recently, some researchers have focused on the function of cellular immunity in the development of tumor. The biological role of IL-33 in glioma is poorly understood. In this study, we showed that glioma cells and tissues expressed higher levels of IL-33 and its receptor ST2 compared to normal brain. Clinically, IL-33 expression was associated with poor survival in patients with glioma. Administration of human IL-33 enhanced cell migration, invasion, epithelial to mesenchymal transition and stemness. Anti-ST2 blocked these effects of IL-33 on tumor. Mechanistically, IL-33 activated JNK signaling pathway via ST2 and increased the expression of key transcription factors that controlled the process of EMT and stemness. Moreover, IL-33 prevented temozolomide induced tumor apoptosis. Anti-ST2 or knockdown IL-33 increased the sensitivity of tumor to temozolomide. Thus, targeting the IL-33/ST2 axis may offer an opportunity to the treatment of glioma patients.

## INTRODUCTION

Interleukin-33 (IL-33) is a member of the interleukin-1 family. It was originally described as an inducer of type 2 immune responses [1]. It is located in nucleus and released as an alarmin in response to tissue damage caused by injury or necrosis [2]. IL-33 exerts its biological functions through binding and activating with its receptor ST2 which has been described as a negative regulator of Toll-like receptor-IL-1 signaling [3]. The IL-33/ST2 axis is widely perceived as the activator of nuclear factor-κB (NF-κB) and mitogen-activated protein kinase (MAPK) pathway including ERK1/2, p38 and JNK [4]. Moreover, IL-33 also promotes the release of proinflammatory cytokines and chemokines including IL-1, IL-6, tumor necrosis factor (TNF) and CCL2 [5]. Both the activated signals and induced cytokines are implicated in tumor development and progression. In line with this, the underlying molecular mechanisms and the potential effect of IL-33 on tumor development remain to be evaluated.

Glioma is the most common malignant brain tumor in human. Currently, clinical first-line treatment of glioma is surgery combined with anti-neoplastic treatment including temozolomide (TMZ) and radiotherapy [6]. The characteristics of high heterogeneity, invasion and drug resistant of glioma make this disease essentially incurable [7]. The mesenchymal phenotype is the hallmark of tumor aggressiveness in human malignant glioma [8]. Functional implications of epithelial-to-mesenchymal transition (EMT) include enhanced mobility, invasion and resistance to apoptotic stimuli [9]. Moreover, tumor cells acquired stemness and chemoresistance properties through EMT [10]. Suppression of EMT increased cancer cell proliferation, enhanced expression of nucleoside transporters and sensitivity to chemotherapy treatment [11]. Thus, effective strategies to reverse mesenchymal phenotype and reduce the resistance to TMZ are urgently needed.

Accumulating evidence demonstrated the relationship between the IL-33/ST2 axis and carcinogenesis. The expression of serum IL-33 was correlated with poor prognosis in gastric cancer, lung cancer and hepatocellular carcinoma [12–14]. IL-33 facilitated proliferation of colorectal cancer dependent on upregulating COX2 expression through NF-κB signaling [15]. Soluble ST2 inhibited colorectal cancer malignant growth by modifying the tumor microenvironment [16]. IL-33/ST2 axis promoted epithelial cell transformation and breast tumorigenesis via upregulation of COT activity [17]. Thus, understanding the role of IL-33 in tumor regulation may provide valuable information for controlling the malignant behavior of glioma. The role of IL-33 in the human brain is under debate. Recent study indicated that IL-33 was produced by astrocytes and mediated synapse numbers and neural circuit function [18]. Moreover, tissue damage including spinal cord injury, stroke and Alzheimer disease promoted IL-33 releasing from nuclear as a cellular alarmin [19–21]. These studies revealed the physiological and pathological functions of IL-33 in central nervous system. Recently, researchers found that overexpression of IL-33 was associated with poor prognosis of patients with glioma [22, 23] and enhanced the tumorigenic activity of rat glioma cells [24]. Furthermore, IL-33 was involved in the process of glioma cell invasion and migration by upregulating MMP2 and MMP9 [25]. However, how IL-33 regulates EMT and stemness of glioma need to be further investigated.

In this research we examined the expression of IL-33 was significantly increased in glioma tissues and cell lines. It also had a significant clinical relationship with the survival of glioma patients. Administration of recombinant human IL-33 promotes glioma cell motility, invasion, EMT and stemness. Glioma cells expressed functional IL-33 receptor ST2. IL-33 activated JNK signal pathway via ST2. Blocking ST2 inhibited the effect of IL-33 on promoting stemness and EMT. In addition, IL-33 prevented chemotherapy-induced tumor apoptosis. Blocked ST2 or knockdown IL-33 enhanced temozolomide induced anti-tumor effects. We believe that IL-33/ST2 axis may be served as a therapeutic target in the treatment of glioma.

## RESULTS

### Expression of IL-33 and ST2 was increased in glioma and correlated with patient prognosis

**Figure 1:**
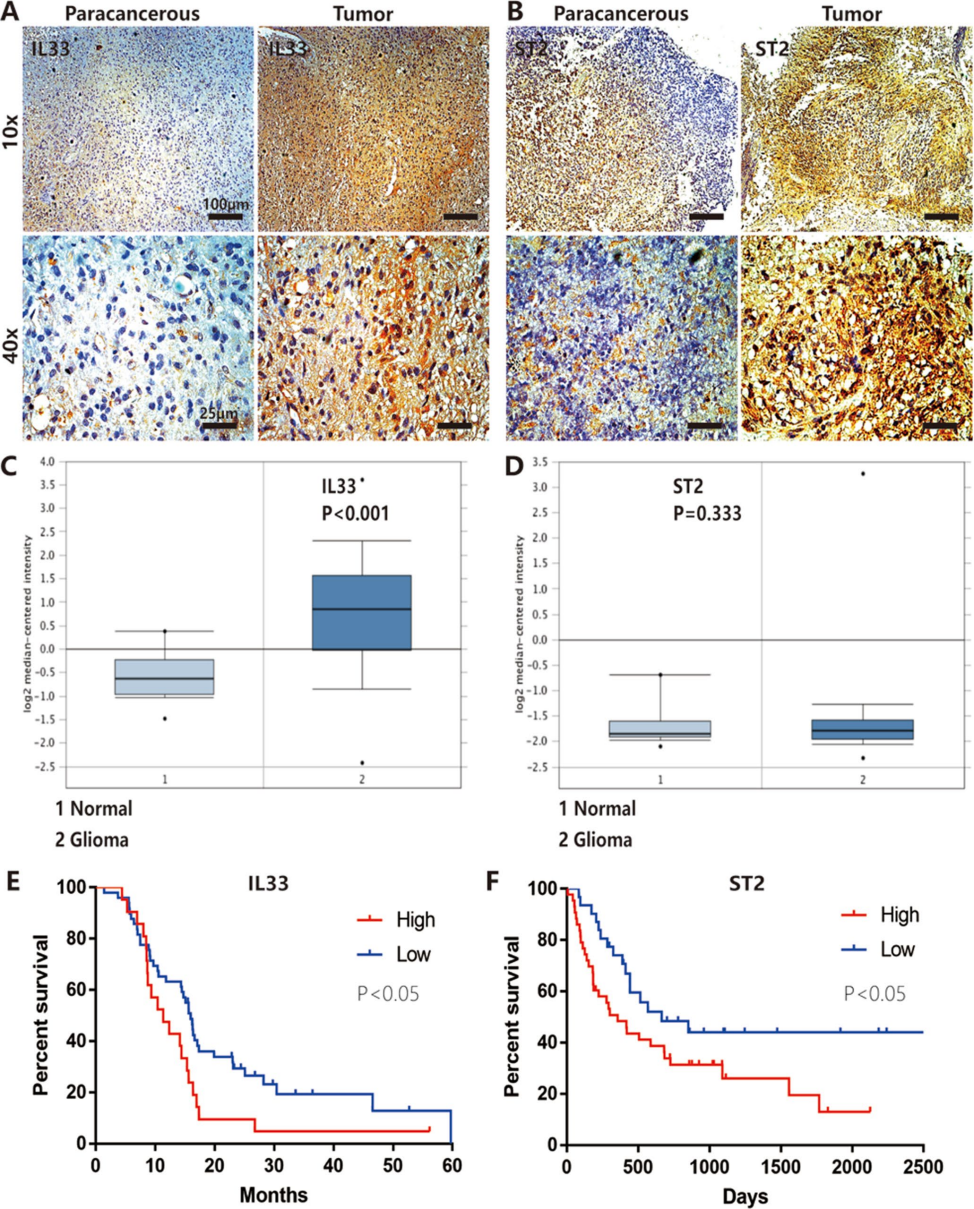
**IL-33 and ST2 expression was increased in glioma and correlated with patient prognosis.** (**A** and **B**) IL-33 and ST2 expression was detected with conventional immunohistochemical staining in clinical glioma samples. The representative images showed IL-33 or ST2 expression was increased in tumor tissues compared with paracancerous tissues. (**C** and **D**) The mRNA expression data of glioma compared with normal brain tissues in the TCGA Database (n=552), the expression of IL-33 was significantly increased in tumor tissues (p<0.001) while ST2 was upregulated moderately with no statistical significance (p=0.333). (**E** and **F**) The association between the survival in patients with glioma and IL-33/ST2 expression (n=66 and n=74 for (**E** and **F**) respectively). Survival functions were estimated by Kaplan–Meier methods. Hazard ratios (HR) for (**E**) High/Low=1.921, Low/High=0.575; HR for (**F**) High/Low=1.828, Low/High=0.547.

We first examined the expression of IL-33 and ST2 in glioma patients of our hospital by immunohistochemistry (IHC). We found that IL-33 and ST2 were heterogeneously expressed in tumor tissues but expressed very low level in paracancerous tissues (Figure 1A and 1B). Next, we analyzed the mRNA expression data of glioma compared with normal brain tissues in the TCGA Data Portal (https://gdc.cancer.gov/), the expression of *IL-33* was significantly increased in tumor tissues while ST2 upregulated moderately with no statistical significance (Figure 1C and 1D). To evaluate the clinical relevance of IL-33 and ST2, we analyzed the survival curves of glioma samples from TCGA database. Kaplan-Meier survival analyses showed that glioma patients with high expression levels of *IL33* had shorter overall survival than those with low levels, the result of ST2 expression was the same (Figure 1E and 1F). These data suggested that IL-33 and ST2 expression might be clinically important in glioma development and progression.

### IL-33 promotes glioma cell migration, invasion and mesenchymal transition

**Figure 2:**
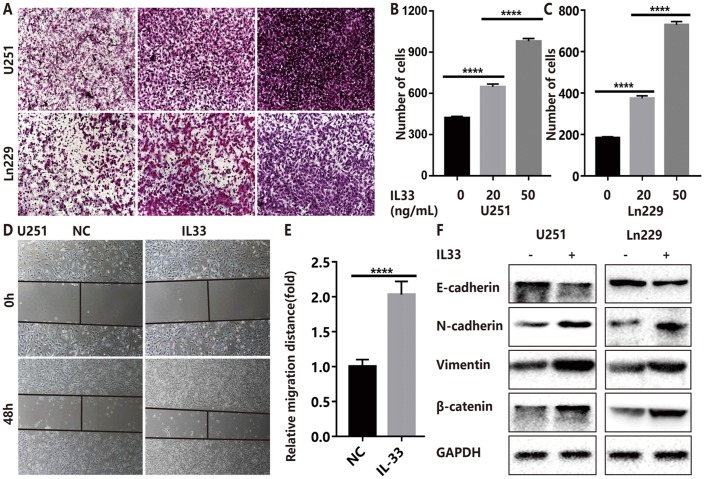
**IL-33 promotes glioma cell invasion, migration and mesenchymal transition.** (**A**) Effects of IL-33 on glioma cells invasion. Glioma cell lines U251 and Ln229 were subject to transwell assay for 24 hours. IL-33 was added in different concentrations. (**B**, **C**) The number of cells moved through the chambers were counted for per field of view and analyzed. Each column represents three independent experiments; Results are expressed as the mean±SD; n=3; ***, P < 0.001; ****, P < 0.0001. (**D**–**E**) IL-33 promotes the migration of glioma cells U251 by wound healing assay. The tumor cells moving distance was detected and divided by control group as relative migration distance for statistical analysis. Results are expressed as the mean±SD; n=3; ****, P < 0.0001. (**F**) The levels of core epithelial marker E-cadherin and mesenchymal markers including vimentin, N-cadherin and β-catenin were detected by Western blotting.

To assess the role of IL-33 in tumor invasion and metastasis, we used two glioma cell lines U251 and Ln229 incubated with or without IL-33. The transwell invasion assays indicated that the number of invasive cells of IL-33 treated group was significantly higher than that of control group (Figure 2A). In addition, we found that IL-33 induced invasive numbers in a dose-dependent manner. High concentrations of IL-33 could stimulate more tumor cells to pass through the chambers, and the results were statistically significant in both two cell lines (Figure 2B and 2C). Wound healing assays showed significant increase in moving distance when treated with IL-33 compared to the control group in both U251 and Ln229 (Figure 2D–2E and Supplementary Figure 1A, 1B). Furthermore, we found that IL-33 promoted epithelial to mesenchymal transition in glioma by detecting the expression of core epithelial and mesenchymal biomarkers. We observed reduced E-cadherin level but increased mesenchymal features such as vimentin, β-catenin and N-cadherin expression (Figure 2F). Thus, IL-33 promotes glioma cell migration, invasion and epithelial to mesenchymal transition.

### IL-33 activates glioma stemness

**Figure 3:**
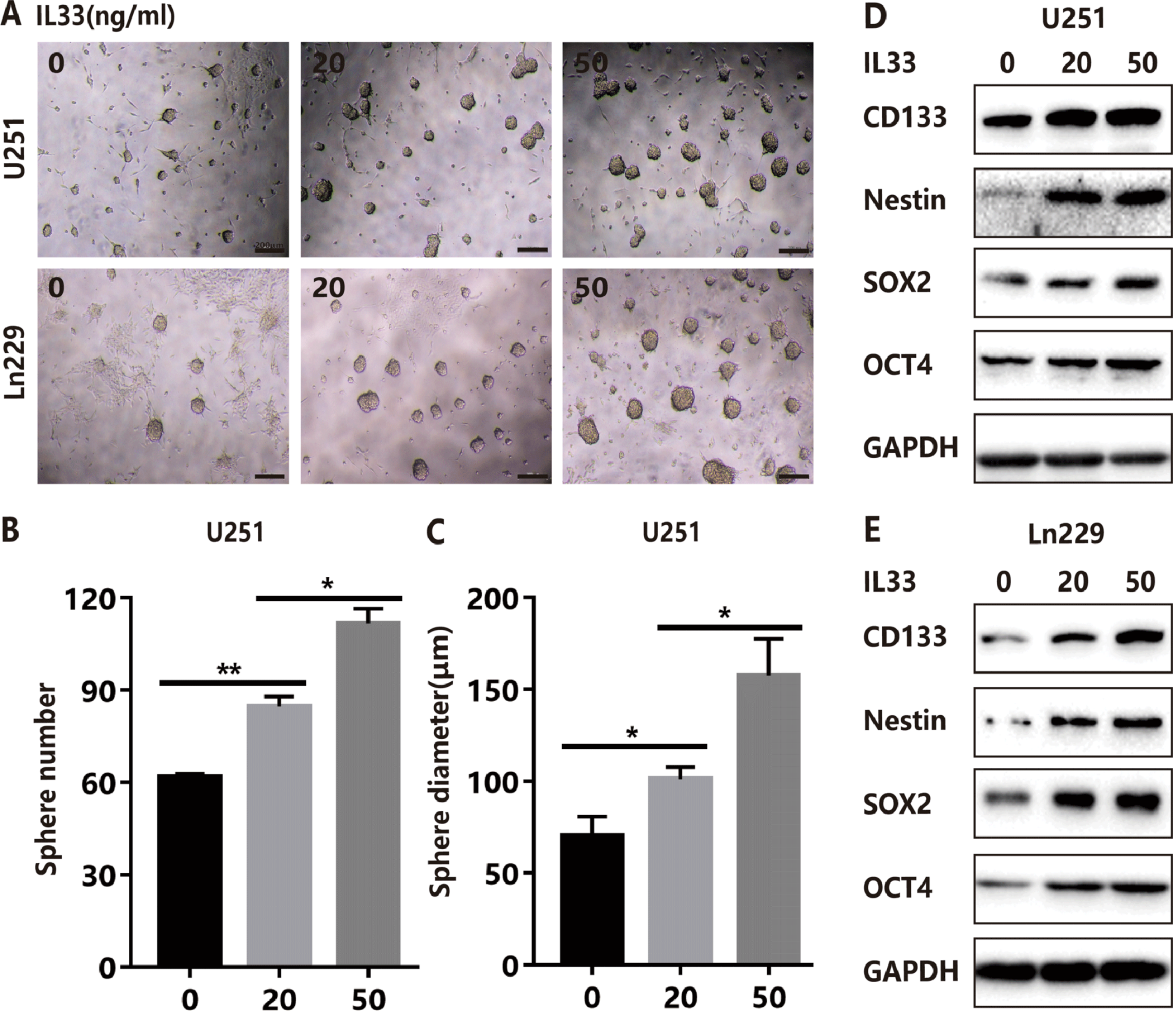
**IL-33 promotes glioma stemness.** (**A**) Effects of IL-33 on glioma cells stemness. Glioma cell lines U251 and Ln229 were subject to sphere formation assay for 7 days. IL-33 was added in different concentrations. Representative images of spheres of glioma cells are shown. Scale bar, 200 μm. (**B** and **C**) The mean numbers and diameters of spheres were counted and analyzed. Each column represents three independent experiments; Results are expressed as the mean±SD; n=3; *, P < 0.05; **, P < 0.01. (**D** and **E**) The levels of core stem cell genes including CD133, Nestin, Sox2 and Oct4 were detected by Western blot.

To explore the effects of IL-33 on glioma sphere formation, glioma cell lines U251 and Ln229 were subject to sphere assay for seven days incubated with or without IL-33. The neurosphere medium consisted of DMEM/F12 supplemented with B27, penicillin/streptomycin, and growth factors EGF and FGF as described [26]. We found that IL-33 induced sphere formation of both glioma cell lines (Figure 3A). Both the number and diameter of neuro-spheres were significantly potentiated by stimulation of IL-33 in a dose-dependent manner (Figure 3B, 3C and Supplementary Figure 2A, 2B). Next, we examined activation on core stem cell genes by western blot. In line with sphere formation assay, IL-33 increased the expression of stem related proteins including CD133, Nestin, Oct4, and Sox2 (Figure 3D and 3E). Therefore, IL-33 activated stem cell related genes and enhanced glioma stemness.

### IL-33 promotes glioma EMT and stemness via JNK activation

**Figure 4:**
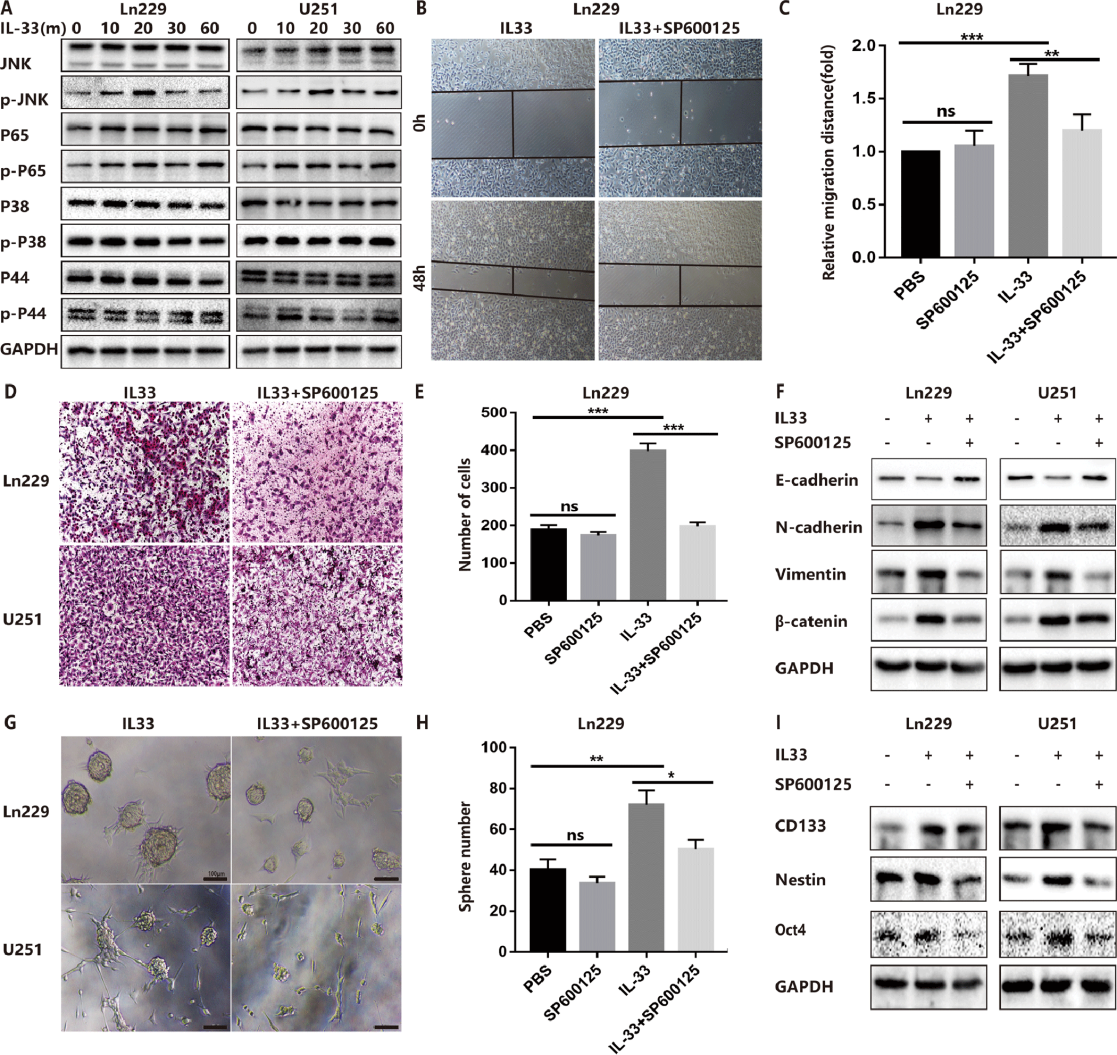
**IL-33 promotes glioma EMT and stemness via JNK activation.** (**A**) Effects of IL-33 on NF-κB and MAPK family signal in glioma cells. The cells were treated with IL-33 (20 ng/mL) in different periods of time. The expression of phosphorylated and total proteins was detected by Western blot. (**B**) Effects of the JNK inhibitor SP600125 on IL-33 induced migration by Wound healing assay. The cells were treated with IL-33 (20 ng/mL) and/or SP600125 (10 μg/mL) for 48 hours. (**C**) The tumor cells moving distance was detected. Experimental group (IL-33, SP600125 and IL-33+SP600125)/Control group were calculated for statistical analysis. Results are expressed as the mean ± SD; n=3; **, P < 0.01; ***, P < 0.001. (**D**) Transwell assay indicated the effects of SP600125 on IL-33 induced invasion. (**E**) The cells moved through the chambers from four groups (PBS, SP600125, IL-33 and IL-33+SP600125) were counted and analyzed. Results are expressed as the mean ± SD; n=3; ***, P < 0.001. (**F**) Effects of the JNK inhibitor SP600125 on EMT related proteins in glioma cells. N-cadherin, E-cadherin, Vimentin and β-catenin proteins were detected by Western blotting. (**G**) Effects of JNK inhibitor on glioma cells stemness. Glioma cell lines were subject to sphere formation assay. Representative images of spheres of glioma cells are shown. Scale bar, 100 μm. (**H**) Sphere number from four groups were counted and analyzed. Results are expressed as the mean ± SD; n=3; *, P < 0.05; **, P < 0.01. (**I**) The expression of CD133, Nestin and Oct4 were detected by Western blot.

The molecular mechanisms of how IL-33 regulates glioma invasion, stemness and mesenchymal transition need to be further investigated. Research proved that IL-33 was the activator of transcription factor p65 (NF-κ B) and the mitogen-activated protein (MAP) kinases including p44/42(ERK), JNK(SAPK) and p38 MAPK [1]. Therefore, we examined the effect of IL-33 on these signaling pathways in glioma cells at different time points. We detected significant activation of the JNK signaling pathway by treated with IL-33 (Figure 4A). The expression of phosphorylated JNK was increased as time went on and reached highest level at 20 minutes. Next, we examined the effect of JNK inhibitor SP600125 on IL-33-induced migration, EMT and stemness. Treated with JNK inhibitor blocked the effect of IL-33 on promoting cell migration (Figure 4B, 4C and Supplementary Figure 3A, 3B) and invasion in both glioma cell lines (Figure 4D, 4E and Supplementary Figure 3C). In line with this, SP600125 reversed the IL-33 induced mesenchymal transition (Figure 4F). Moreover, JNK inhibitor blocked glioma sphere formation (Figure 4G). Sphere numbers were decreased significantly (Figure 4H and Supplementary Figure 3D). The IL-33 induced stem cell core gene expression also blocked by JNK inhibition (Figure 4I). Therefore, IL-33 promotes glioma cells migration, invasion, EMT and stemness via JNK activation.

### IL-33 interacts with ST2 to activate JNK-enhanced EMT and stemness

**Figure 5:**
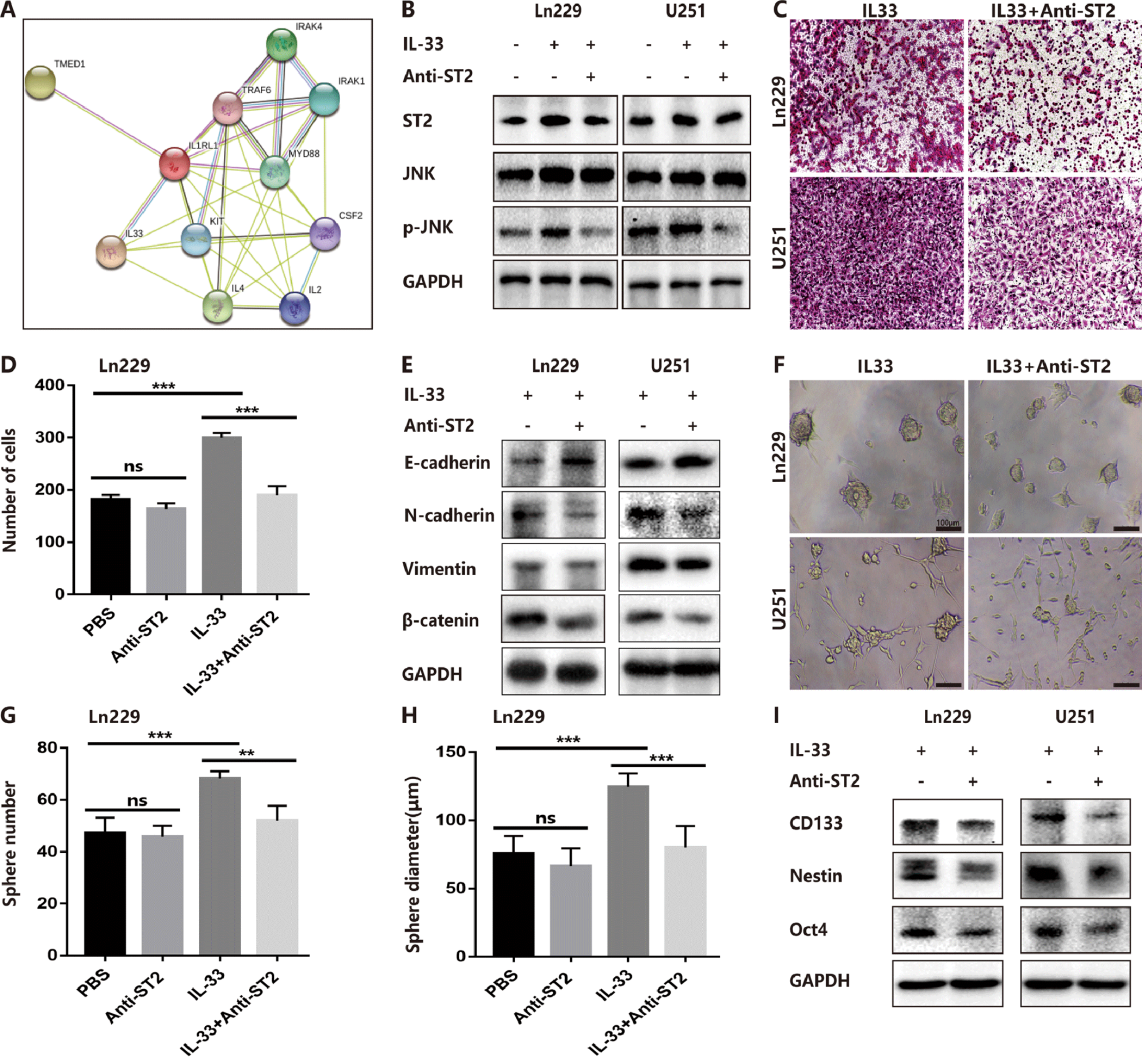
**IL-33 interacts with ST2 to activate JNK-enhanced invasion, EMT and stemness.** (**A**) IL-33 and ST2 interaction network from string. (**B**) Effects of IL-33 on ST2 expression and anti-ST2 blocked the IL-33-induced JNK activation by western blot. (**C**) Effects of anti-ST2 on the role of IL-33-induced glioma invasion. Glioma cell lines were subject for transwell assay. (**D**) IL-33 (20ng/mL) and/or anti-ST2 antibody (1 ug/mL) were added in the cell culture. Results are expressed as the mean ± SD; n=3; ***, P < 0.001. (**E**) Effects of anti-ST2 on the role of IL-33-stimulated EMT related protein expression. Glioma cells were cultured with IL-33 (20 ng/mL) and/or anti-ST2 antibody (1 ug/mL) for 48 hours. The expression of N-cadherin, E-cadherin, Vimentin and β-catenin were quantified by western blot. (**F**) Effects of anti-ST2 on the role of IL-33-induced glioma stemness. Anti-ST2 blocked the IL-33-induced sphere formation. Representative images of spheres of glioma cells are shown. Scale bar, 100 μm. (**G**–**H**), The mean numbers and diameters of spheres were counted and analyzed. Results are expressed as the mean ± SD; n=3; **, P < 0.01; ***, P < 0.001. (**I**) The levels of stemness related genes including CD133, Nestin and Oct4 were detected by Western blot. Results are shown as mean of the data from at least three independent experiments.

ST2, also known as IL-1R1, was the receptor of IL-33 that was first identified in oncogene- or serum-stimulated fibroblasts. As shown in string network (https://string-db.org/), IL-33 and ST2 had high level interaction (Figure 5A). We had confirmed the expression of ST2 was increased in human gliomas compared with normal tissues (Figure 1B) and IL-33 treatment increased ST2 expression in glioma cells (Figure 5B). To assess whether IL-33 activated downstream signals via binding with ST2, we blocked ST2 with neutralizing mAb against ST2 (anti-ST2 mAb). We found that IL-33 activated JNK signal was blocked by anti-ST2 (Figure 5B). Treated with neutralizing mAb against ST2 blocked the effects of IL-33 in glioma cells invasion (Figure 5C, 5D). In addition, the IL-33 induced epithelial to mesenchymal transition could be reversed by anti-ST2 antibody. The expression of mesenchymal markers was decreased, while epithelial marker was increased (Figure 5E). Furthermore, anti-ST2 blocked the effects of IL-33 in glioma sphere formation (Figure 5F). Sphere number and diameter was decreased in glioma cell line (Figure 5G, 5H). The expression of core stem cell proteins was inhibited by anti-ST2 (Figure 5I). Thus, ST2 is expressed by glioma cells and mediates IL-33 downstream signaling.

### Blocked IL-33/ST2 axis enhances Temozolomide induced anti-tumor effects

**Figure 6:**
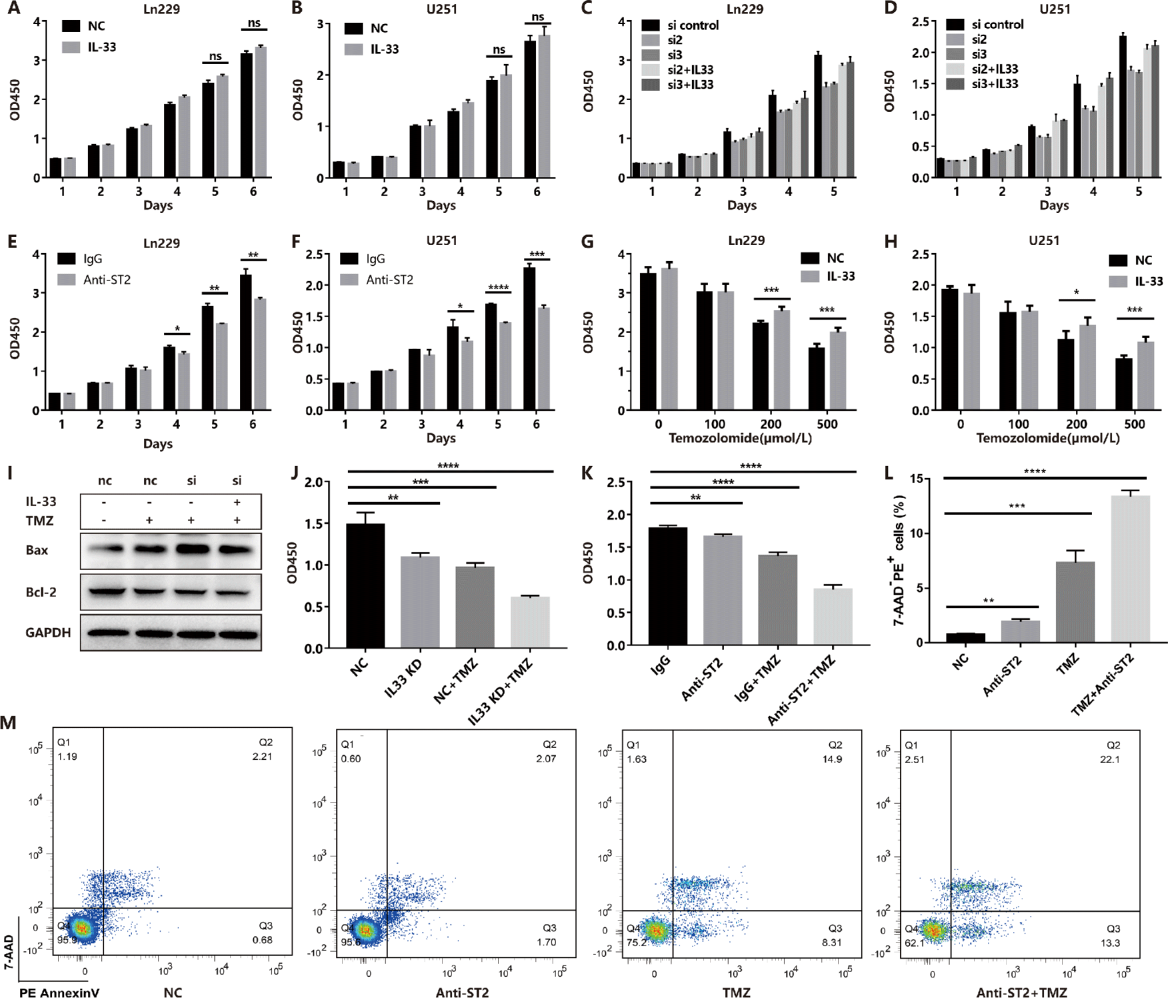
**IL-33 prevents TMZ-induced apoptosis and blocked IL-33/ST2 increases tumor apoptosis.** (**A** and **B**) Effects of IL-33 on proliferation of glioma. Glioma cell lines were cultured with or without IL-33 (20 ng/mL) for 6 days. The cell viability was determined by CCK-8 assay. (**C** and **D**) Effects of si-IL-33 on glioma cells proliferation. Si-IL-33 and control si-RNA expressing glioma cells were cultured with or without IL-33 (20 ng/mL) for 5 days. The cell viability was determined by CCK-8. (**E** and **F**) Effects of anti-ST2 on glioma cells proliferation. Glioma cell lines Ln229 and U251 were treated with anti-ST2 antibody (1 ug/mL) or IgG control for 6 days. The cell viability was determined by CCK-8. (**G** and **H**) Effects of IL-33 on glioma chemotherapy. Glioma cell lines Ln229 and U251 were cultured with or without IL-33 (20 ng/mL) for 24 hours and were subsequently exposed to TMZ for 48 hours. The cell viability was determined by CCK-8 assay. (**I**–**K**) IL33 was knocked down by si-IL-33 and si-control, the si and control groups were treated with IL-33 (20 ng/mL) or/and TMZ (200uM). The expression of Bax, Bcl-2 were detected by western blot (**I**) and the cell viability was determined by CCK-8 (**J** and **K**). (**L**–**M**) We performed the Annexin V/PI staining to measure apoptosis. Glioma cells were treated with PBS, anti-ST2, TMZ and anti-ST2+TMZ. Percent of 7-AAD^-^PE^+^ cells were counted for analysis. All results are expressed as the mean ± SD from at least three independent experiments; n=4; *, P < 0.05; **, P<0.01; ***, P<0.001; ****, P<0.0001.

Although we proved the role of IL-33 in promoting stemness and EMT, we did not observe a significant effect on glioma proliferation in vitro (Figure 6A and 6B). However, we genetically knocked down IL-33 with specific siRNA (Supplementary Figure 4A) and found that si-IL-33 reduced the proliferation of glioma cells (Figure 6C and 6D). Addition of recombinant IL-33 reversed the inhibition of proliferation (Figure 6C and 6D). Interestingly, anti-ST2 treatment inhibited tumor proliferation (Figure 6E and 6F). We speculated that glioma cells may release high level IL-33 that resulted in the stimulation of exogenous IL-33 for proliferation was not significant. We hypothesized that IL-33 may induce the TMZ chemoresistance as its role in promoting EMT and stemness in glioma. Glioma cells were cultured with or without IL-33 and were subsequently exposed to TMZ in different concentrations. The results showed that IL-33 reduced the anti-tumor effect of TMZ (Figure 6G and 6H). We found that TMZ increased the expression of apoptosis related protein Bax when knockdown IL-33. However, the expression of anti-apoptotic protein Bcl-2 did not change obviously (Figure 6I and Supplementary Figure 4B). Both IL-33 knockdown or anti-ST2 treatment enhanced the anti-tumor effect of TMZ (Figure 6J and 6K). We adopted the Annexin V/PI staining to measure apoptosis. The result revealed that anti-ST2 treatment enhanced the TMZ-induced apoptosis (Figure 6L and 6M). Thus IL-33/ST2 axis may be served as a therapeutic target in combination with TMZ chemotherapy in glioma treatment.

### In vivo experiments prove the effect of IL-33 on mesenchymal transition, stemness and tumorigenesis

**Figure 7:**
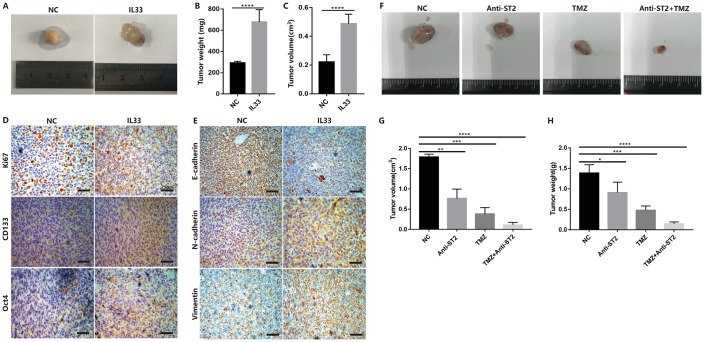
**IL-33 promotes glioma tumorigenesis, EMT and stemness in vivo.** (**A**) Glioma cells (Ln229) were subcutaneously injected into nude BALB/c mice in the presence or absence of 1ug/ml IL-33 and allowed to grow until tumors formed (4 weeks). (**B** and **C**) The tumor volume and tumor weight were monitored at 4 weeks after injection. Results are expressed as the mean of tumor volume ± SD; n=5; ****, P<0.0001. (**D** and **E**) primary glioma sections form mouse models were stained with proliferative maker Ki67, EMT related antibodies (E-cadherin, N-cadherin and Vimentin) and core stem genes (CD133 and Oct4) for IHC assay. (**F**) Glioma cells were subcutaneously transplanted into back flanks of NSG mice. After transplanted for 3 weeks, PBS, Anti-ST2, TMZ and Anti-ST2+TMZ were administered intraperitoneally (i.p.) into mice for consecutive 7 days. All NSG mice were killed in the sixth week. (**G** and **H**) tumor volume and weight were monitored and analyzed. Results are expressed as the mean of tumor volume ± SD; n=5; *, P < 0.05; **, P<0.01; ***, P<0.001; ****, P<0.0001.

To examine the role of IL-33 in glioma tumorigenesis, EMT and stemness in vivo, we used glioma cell line Ln229 that subcutaneously injected into nude mice with or without IL-33. The mouse models were intraperitoneal injected with IL-33 or PBS six times in the first two weeks and allowed tumors formation until four weeks. Representative tumor images demonstrated that IL-33 significantly induced glioma development (Figure 7A). The tumor weight (Figure 7B) and tumor volume (Figure 7C) were monitored and analyzed. Glioma sections from mouse models were stained with Ki67, EMT and stemness related antibodies for Immunohistochemistry assay. The result indicated that expression of Ki67 was increased when treated with IL-33 (Figure 7D). That meant IL-33 had a certain role in promoting glioma proliferation in vivo. The IL-33 treated group showed a high-level expression of the core stem cell markers including CD133 and Oct4 (Figure 7D). Treated with IL-33, the expression of E-cadherin was decreased while the expression of vimentin and N-cadherin was increased (Figure 7E). To further exclude the effect of IL-33 on mast cells and ILC2, we used the NSG mouse which lack mast cells and ILC2. We set the experiment that we divided the mice into four groups including Negative control, Anti-ST2 treatment, TMZ treatment and TMZ+Anti-ST2 treatment group. Glioma cells were subcutaneously transplanted into back flanks of NSG mice. After transplanted for 3 weeks, PBS, Anti-ST2, TMZ and Anti-ST2+TMZ were administered intraperitoneally (i.p.) into mice for consecutive 7 days. All NSG mice were killed in the sixth week. We found that Anti-ST2+TMZ significantly reduced glioma development (Figure 7F). The tumor volume and weight (Figure 7G and 7H) were monitored and analyzed.

## DISCUSSION

Glioblastoma is the most aggressive type of glioma that just has a median survival of 15 months [27]. Research found that mesenchymal glioma stem cells displayed more aggressive phenotypes in vitro and were markedly resistant to radiation [28, 29]. Although the introduction of temozolomide has advanced improvement for the glioma chemotherapy, development of chemoresistance is still the major hurdle in cancer treatment. Emerging evidence demonstrated molecular and phenotypic association between EMT and chemoresistance in several cancers. After chemotherapy the residual breast cancers displayed a mesenchymal phenotype and stem cell features [30]. Studies indicated that EMT cells significantly contributed to recurrent tumor formation after chemotherapy. These cells survived antineoplastic treatment due to reduced proliferation, apoptotic tolerance and increased expression of chemoresistance-related genes [11, 31]. In addition to enhance the ability of cell invasion properties, EMT was also associated with a stem cell-like behavior that made the treatment more difficult [32]. Our study demonstrated that mesenchymal transition in glioma promoted tumor invasion and migration. EMT cells had the characteristics of resistance to TMZ treatment. Effective therapies against strong ability of EMT induced invasion and chemoresistance appear to be crucial for increasing survival and improving the quality of life.

Cytokines of the interleukin-1 family have pleiotropic activities in innate and adaptive immune responses in host defense, immune regulation, and inflammation. IL-33 is an important member of the IL-1 family. It is a ligand of the ST2 receptor, a member of the IL-1 receptor family. Binding with ST2, IL-33 mediates its biological effects by activating MAP kinases and NF-κB and promotes production of cytokines from polarized TH2 cells [1]. Recently, more attention has been paid to the role of the IL-33/ST2 axis in tumorigenesis and progression. Study indicated that IL-33 promoted colon cancer cell stemness via JNK activation and macrophage recruitment [33]. By upregulated the activity of COT, IL-33/ST2 axis promoted breast tumorigenesis and EMT [17]. In central nervous system, developing astrocytes produced amounts of IL-33. The astrocyte induced IL-33 promoted microglial synapse engulfment and drove synapse depletion [18]. The biological role of IL-33 in glioma need to be investigated more deeply. Accumulating studies also demonstrated that the anti-tumor effects of IL-33 mediated by non-tumor cells. IL-33 was originally described as an inducer of type 2 immune responses, activating TH2 cells and mast cells. As evidence becomes accumulating, IL-33 also potently stimulates group 2 innate lymphoid cells (ILC2s), regulatory T (Treg) cells and other T cells. Research demonstrated that IL-33 promoted the formation of a pro-tumorigenic microenvironment by enhancing the accumulation of mast cells [34]. On the other hand, IL-33 may also limit tumor growth in other conditions, through the activation of cells involved in anti-tumor immunity by activating NK cells and CD8^+^ T cells [35].

Here, we demonstrated that IL-33 was a critical tumor promoter during epithelial to mesenchymal transition and stemness in glioma. We found that IL-33 and ST2 expression was significantly increased in glioma cells and tissues. As has been described previously demonstrating no nuclear staining of IL-33 in normal brain tissues but strong nuclear staining in glioblastoma by Gramatzki [22]. We analyzed the mRNA expression data from TCGA database and found the expression of *IL-33* was significantly increased in glioblastoma tissues compared with normal brain. Previous study also found that the median IL-33 mRNA expression levels varied significantly but multiple comparisons between each tumor grade did not demonstrate statistically significant results [22]. Administration of human recombinant interleukin-33 could activate the JNK signal pathway in glioma. Treated with JNK inhibitor we found a reversed EMT transition and reduced sphere formation. Anti-ST2 blocked these effects of IL-33 on glioma cells. IL-33 binding with its receptor ST2 promoted EMT and stemness in glioma via JNK activation. Interestingly, we did not detect the significant role of IL-33 in promoting proliferation in vitro. However, both IL-33 knockdown and Anti-ST2 antibody significantly reduced the proliferation of glioma cells. We suspected that the glioma cells themselves might express and release large amounts of IL-33, which made the stimulation of exogenous IL-33 insignificant. Or because of the changes of tumor microenvironment which could not be simulated in vitro leads to the weak effect of IL-33. Our in vivo experiments proved that IL-33 significantly induced glioma development in nude mice. To further exclude the effect of IL-33 on the mast cells and ILC2, we used the NSG mice which lack mast cell and ILC2. We observed that anti-ST2 treatment suppressed tumor growth. Furthermore, combined application of anti-ST2 and TMZ produced more significant inhibition on tumor growth. However, our in vivo experiments still had limitations that we did not completely exclude the effect of IL-33 on the mast cells and ILC2. Using the ST2 knockout mice and ST2-KO cell line could better exclude the effect of IL-33 on the immune system.

After surgical resection, adjuvant chemotherapy and radiotherapy are regarded as the critical component of the current standard care for the residual invasive glioma cells. Understanding the biological mechanism of radio- or chemo- resistant in glioma is critical due to the exceedingly poor prognosis. Here we found that the inhibitory effect of TMZ on tumor cell proliferation could be reduced by treating with IL-33. Knockdown IL-33 or blocked the ST2 with anti-ST2 antibody in combination with TMZ decreased the proliferation of glioma cells more significantly. Chemoresistance is still a major obstacle in the treatment of gliomas. Cancer stem cells have a subpopulation that is highly tumorigenic and has high ability of self-renew which makes tumors resistant to various therapies [36]. Because of IL-33 in promoting EMT and stemness in glioma, the resistant to TMZ caused by IL-33 might be mediated by these biological changes. It is also consistent with the reports that EMT contributes to chemoresistance in pancreatic cancer and breast cancer [11, 31]. Our work suggests that targeting IL-33 and ST2 signaling may be potentially applicable in targeting EMT and stemness treatment and enhancing the sensitiveness of chemotherapies, for glioma treatment.

## MATERIALS AND METHODS

### Tumor cell lines

Human glioma cell lines, U251 and Ln229 were purchased from the Chinese Academy of Sciences Cell Bank. All the cell lines were cultured in a 5% CO2 atmosphere at 37 degree and maintained with Dulbecco’s Modified Eagle’s Medium (DMEM) supplemented with 10% fetal bovine serum (FBS). DMEM was purchased from Corning and FBS was purchased from Sigma.

### Glioma patients and tissues

Patients diagnosed with glioma were recruited in the study. All usage of human subjects in this study was approved by the local Institutional Review Board. Formalin-fixed, paraffin-embedded glioma tissue blocks (n=30) were obtained during surgery. Among them 22 cases were WHO grade IV and 8 cases were WHO grade II-III. These patients underwent resection at the second affiliated hospital of HMU. Microarray data were obtained from The Cancer Genome Atlas (TGCA, http://cancergenome.nih.gov/). The reporters used in TCGA database were 209821_at for *IL-33* and 207526_s_at for *IL1RL1*. The TCGA database used for this study contains data of 542 glioblastoma samples and 10 normal brain samples.

### Western blotting

Western blotting was performed with specific antibodies against human. All antibodies were used at the dilution according to manufacturer including anti-ST2 (HPA007917; Sigma), anti-IL-33 (Nessy-1; Enzo) and anti-GFAP (A14673; ABclonal), anti-POU5F1 (A7920; ABclonal), anti-Sox2 (A0561; ABclonal), anti-Nestin (19483-1-AP; Proteintech), anti-β-catenin (17565-1-AP; Proteintech) and anti-CD133 (64326; Cell Signaling Technology), anti-E-cadherin (3195; Cell Signaling Technology), anti-N-cadherin (13116; Cell Signaling Technology), anti-Vimentin (5741; Cell Signaling Technology), anti-SAPK (JNK) (9252; Cell Signaling Technology), anti-P38 (8690; Cell Signaling Technology), anti-P44/42 (4695; Cell Signaling Technology), anti-NF-κB (10745-1-AP; Proteintech), anti-p-SAPK (JNK) (4668; Cell Signaling Technology), anti-p-P44/42 (4377; Cell Signaling Technology), anti-p-P38 (4511; Cell Signaling Technology), anti-p-NF-κB (3033; Cell Signaling Technology) and anti-GAPDH (Cell Signaling Technology). Following incubation in HRP labeled secondary antibody (Cell Signaling Technology). Specific protein bands were scanned with ECL detection reagents and detected by Gel Doc 2000 (Bio-Rad). The bands of western blots were quantified by ImageJ and shown in the Supplementary Figure5.

### Wound healing assay

Tumor cells were seeded into the 6-well plates until more than 70 % of confluent growth. After washing by PBS, the medium was replaced by serum-free cell culture. Photographs of the scratch-wound gap were taken at 0 and 48 h by an Axiovert 200 microscope (Carl Zeiss). For each well the scratch was analyzed in triplicate.

### Transwell assay

Invasion assays were carried out using transwell inserts (Corning) according to the manufacturer’s protocol. The Matrigel matrix was diluted in serum-free medium to a final concentration of 200 μg/mL and mixed thoroughly by gently pipetting the matrix up and down. Next, 100 μL of the diluted Matrigel matrix was carefully added to the chamber. The plate was incubated at 37°C for 1 hour to allow the Matrigel matrix to form a gel. The cells were diluted to a density of 5 x 10^4^/200μL with serum-free medium and seeded into the upper chamber of each well. The chambers were then incubated for 24 or 48 h in culture medium with 10% FBS in the bottom. The cells on the upper surface were scraped and washed away and the cells on the lower surface were fixed with methanol for 20 min and then stained with hematoxylin and eosin. Finally, the cell numbers were quantified under a microscope. Four independent fields were counted for each well and repeated in triplicate.

### Sphere formation assay

A tumorsphere is a solid, spherical formation developed from the proliferation of one cancer stem/progenitor cell. These tumorspheres are easily distinguishable from single or aggregated cells as the cells appear to become fused together and individual cells cannot be identified. Cells are grown in serum-free, non-adherent conditions in order to enrich the cancer stem/progenitor cell population as only cancer stem/progenitor cells can survive and proliferate in this environment. This assay can be used to estimate the percentage of cancer stem/progenitor cells present in a population of tumor cells. Glioma cell lines U251 and Ln229 were subject to sphere medium for at least seven days incubated with or without IL-33. The neurosphere medium consisted of DMEM/F12 supplemented with B27, penicillin/streptomycin, and growth factors EGF (20 ng/ml) and FGF (20 ng/ml) as described [26]. Spheres (>50 μm) were counted.

### Immunohistochemistry straining methods

Tissue sections were fixed with formalin and embedded with paraffin. The slices were labeled with anti-human IL-33 and anti-ST2. Immunostaining intensity and reactivity were examined by Case Viewer after scanning using digital microscope. The expression of proteins was quantified by a 4-value score criterion that consisted with intensity score (0, 1, 2, 3 for none, weak, moderate and strong) and percentage score (0, <10%; 1, 10–40%; 2, 40–70%; 3, >70%). The final score was the intensity values plus percentage score. We quantified the staining and analyzed the differences of expression in the Supplementary Figure 6.

### Cell proliferation assay

Cell proliferation was evaluated using the Cell counting kit-8 (CCK-8) assay according to the protocol. Briefly, cells were seeded in 96-well plates and incubated with various concentrations of TMZ (Selleck), IL-33 (Human recombinant murine IL-33, R&D Systems) or Anti-ST2 (Human ST2/IL-33R Antibody, R&D Systems). Subsequently, CCK-8 solution was added, and the viable cells were quantified using IMARK microplate reader at 450 nm of absorbance. Each experiment was performed in triplicate and repeated for three times.

### Flow cytometry apoptosis assay

We performed the flow cytometry to detect the apoptosis of tumor cells. PE Annexin V staining precedes the loss of membrane integrity which accompanies the latest stages of cell death resulting from either apoptotic or necrotic processes. Therefore, staining with PE Annexin V is typically used in conjunction with a vital dye such as 7-Amino-Actinomycin (7-AAD) to allow the investigator to identify early apoptotic cells (7-AAD negative, PE Annexin V positive). Viable cells with intact membranes exclude 7-AAD, whereas the membranes of dead and damaged cells are permeable to 7-AAD. Cells that are considered viable are PE Annexin V and 7-AAD negative; cells that are in early apoptosis are PE Annexin V positive and 7-AAD negative; and cells that are in late apoptosis or already dead are both PE Annexin V and 7-AAD positive. Details protocol PE Annexin V Apoptosis Detection Kit I

### Animal models and in vivo tumor formation

Four to five-week-old female nude BALB/c mice (Beijing HFK Bioscience Co., Ltd) and five to six-week-old female NSG mice (Shanghai Model Organisms) were used for subcutaneous xenograft models. All experiments were approved by the Ethics Committee of Harbin Medical University (HMU, Harbin, Heilongjiang, China) according to the Guidelines for the Care and Use of Laboratory Animals. Ln229 glioma cells were subcutaneously transplanted into back flanks of BALB/c nude mice. After transplanted for 2 weeks, human recombinant murine IL-33 (carrier-free, R&D Systems) or PBS was administered intraperitoneally (i.p.) into mice at 1 μg per injection for 10 days. All nude mice were killed in the fourth week. Ln229 glioma cells were subcutaneously transplanted into back flanks of NSG mice. After transplanted for 3 weeks, PBS, Anti-ST2, TMZ and Anti-ST2+TMZ were administered intraperitoneally (i.p.) into mice for consecutive 7 days. All NSG mice were killed in the sixth week.

### Statistical analysis

We performed unpaired student t-test to compare two groups when appropriate. In cases of multiple groups, statistical analysis was performed through one-way ANOVA analysis using the SPSS software. Survival functions were evaluated by Kaplan–Meier methods. Statistical results with mean ± SEM in the experiments were calculated using GraphPad Prism software (version 7.0). All in vitro results were shown as mean of the data from at least three independent experiments and n in figure legends means technical replicates. A value of P < 0.05 was considered as statistical significance.

## Supplementary Material

Supplementary Figures
