# Supplementary material for: The Interleukin-33/ST2 axis promotes glioma mesenchymal transition, stemness and TMZ resistance via JNK activation
Source: Aging (Albany NY). 2020 Jan 29;12(2):1685–703. doi: 10.18632/aging.102707 (PMC7053587; doi:10.18632/aging.102707)
Supplement: Supplementary Figures [file aging-12-102707-s001..pdf]

SUPPLEMENTARY FIGURES

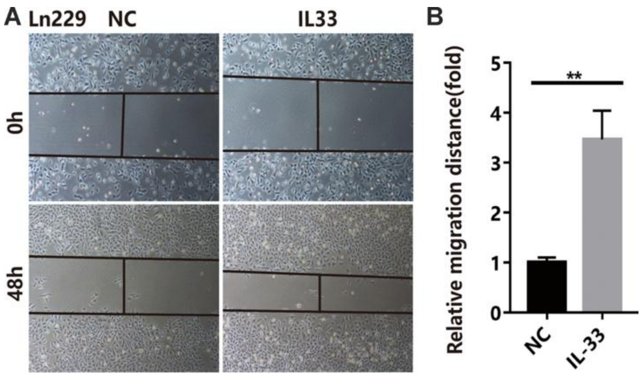

Supplementary Figure 1. (**A** and **B**) IL-33 promotes the migration of glioma cells Ln229 by wound healing assay. The tumor cells moving distance was detected and divided by control group as relative migration distance for statistical analysis. Results are expressed as the mean ± SD; n=3; **, P < 0.01.

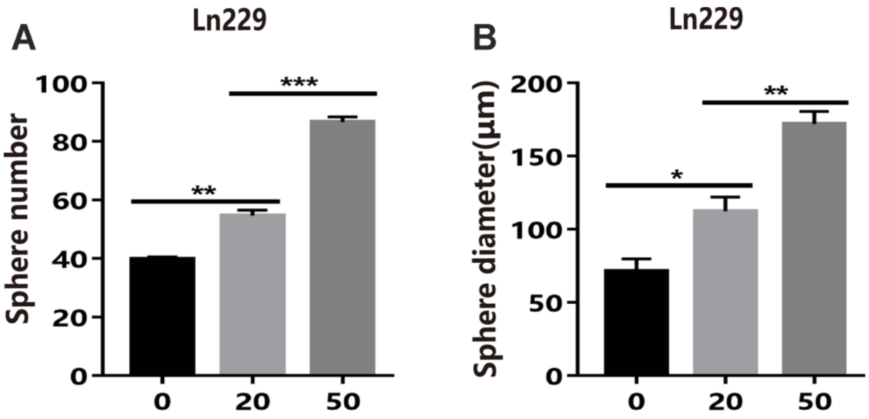

Supplementary Figure 2. (**A** and **B**) numbers and diameters of spheres were counted and analyzed. Each column represents three independent experiments; Results are expressed as the mean ± SD; n=3; *, P < 0.05; **, P < 0.01; ***, P < 0.001.

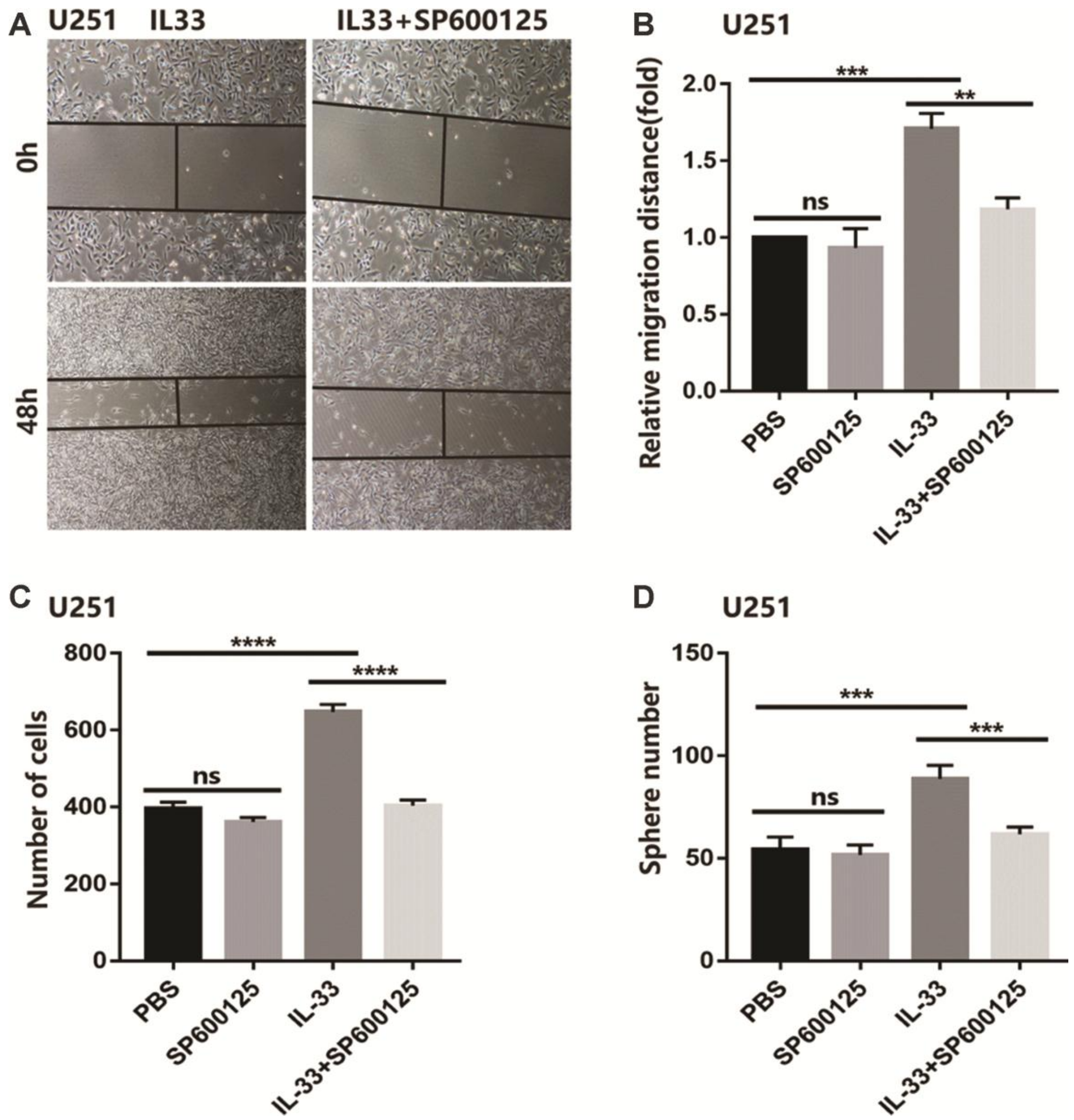

Supplementary Figure 3. (**A** and **B**) Effects of the JNK inhibitor SP600125 on IL-33 induced migration by Wound healing assay in glioma cell line U251. (**C** and **D**) Effects of JNK inhibitor on glioma stemness of U251. The mean numbers and diameters of spheres were counted and analyzed. Each column represents three independent experiments; Results are expressed as the mean ± SD; n=3; **, $P < 0.01$; ***, $P < 0.001$; ****, $P < 0.0001$.

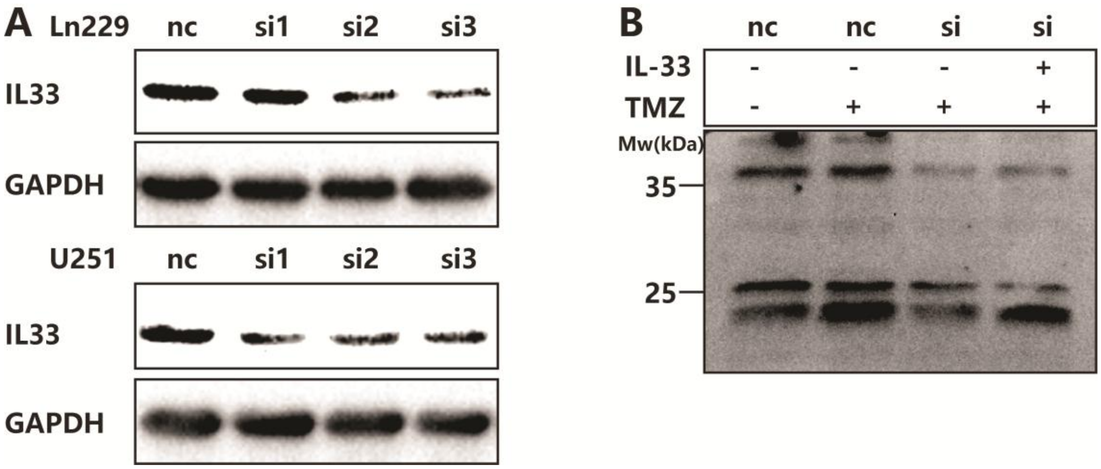

Supplementary Figure 4. (**A**) Glioma cell lines Ln229 and U251 were treated with three si-IL-33 RNA and si-control, the IL-33 protein expression was detected by western blot. (**B**) IL33 was knocked down with si-IL-33 and si-control, the si and control groups were treated with IL-33(20 ng/mL) or/and TMZ (200uM). The expression of full-length and cleaved form of IL-33 were detected by western blot.

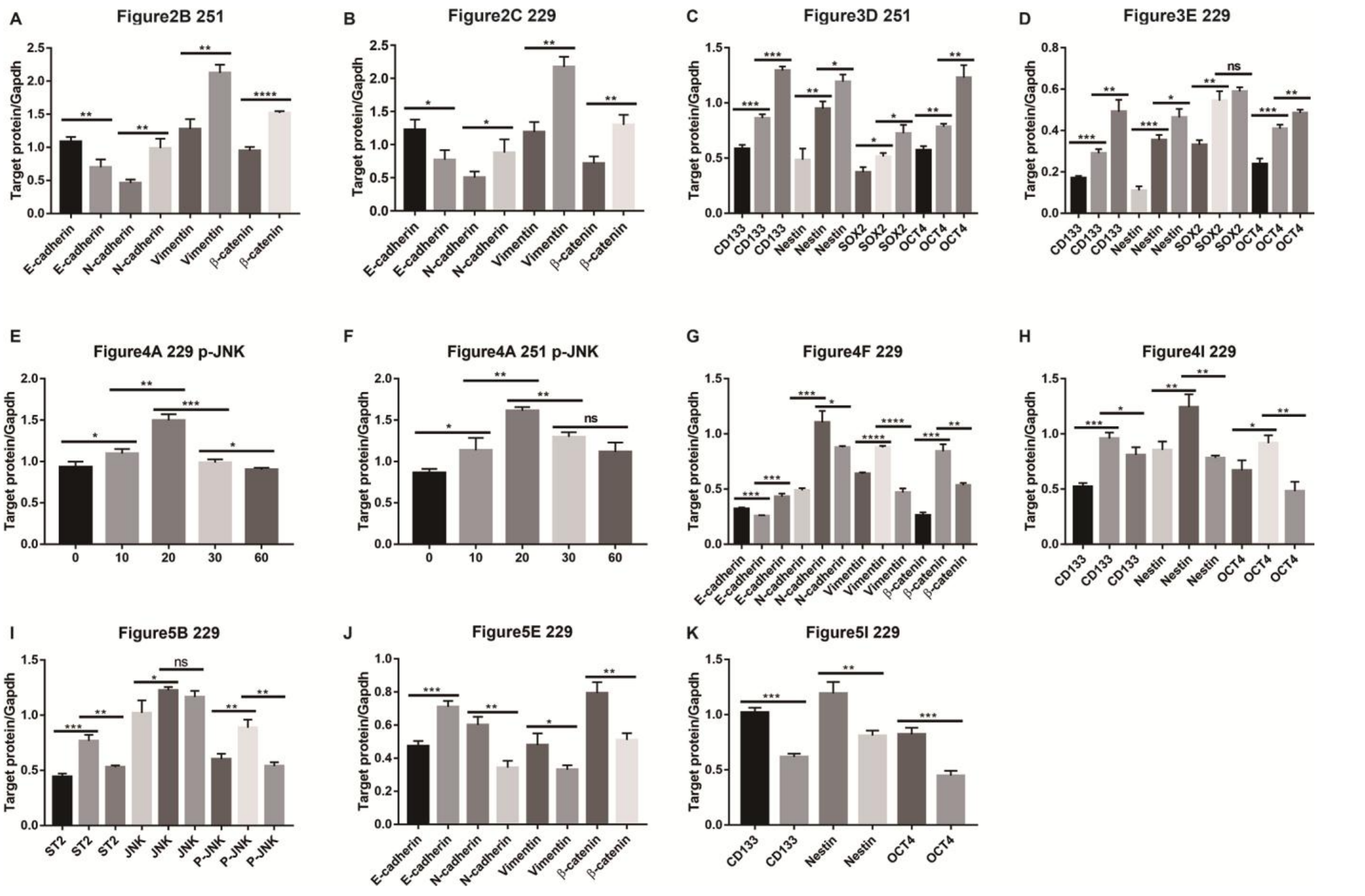

Supplementary Figure 5. The bands of western blots were quantified by ImageJ and analyzed.

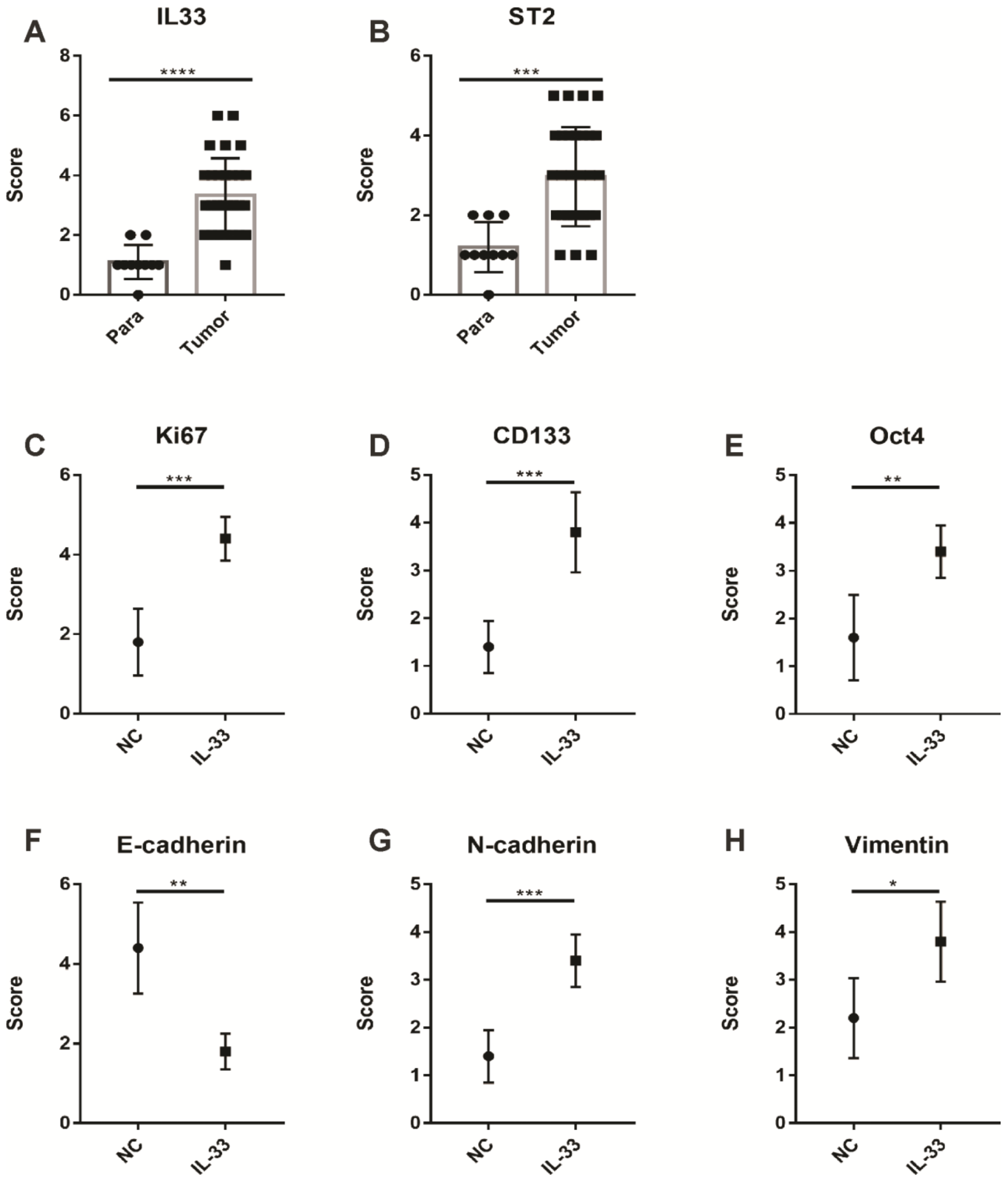

Supplementary Figure 6. (**A** and **B**) we quantified the staining of immunohistochemistry and analyzed the differences of expression for Figure1A and 1B. (**C**–**H**) we quantified the staining of immunohistochemistry and analyzed the differences of expression for Figure7D and 7E.
